# Alginate oligosaccharide supplementation improves boar semen quality under heat stress

**DOI:** 10.1007/s44154-024-00177-7

**Published:** 2024-09-03

**Authors:** Yexun Zhou, Zeou Wei, Jiajian Tan, Haiqing Sun, Haidi Jiang, Yang Gao, Hongfu Zhang, Martine Schroyen

**Affiliations:** 1 State Key Laboratory of Animal Nutrition, Institute of Animal Sciences, Chinese Academy of Agricultural Sciences, Beijing, 100193 P. R. China; 2 Precision Livestock and Nutrition Unit, Gembloux Agro-Bio Tech, University of Liège, Gembloux, Belgium; 3 College of Life Science, Baicheng Normal University, Baicheng, 137000 Jilin China; 4 YangXiang Joint Stock Company, Guigang, 53700 China; 5 School of Agriculture and Food Science, University College Dublin, Belfeld, Dublin 4 Ireland

**Keywords:** Heat stress, Alginate oligosaccharides (AOS), Semen quality, Gut microbiota, Sperm metabolome, Sperm proteome, Boars

## Abstract

Heat stress is a serious problem that affects animal husbandry by reducing growth and reproductive performance of animals. Adding plant extracts to the diet is an effective way to help overcome this problem. Alginate oligosaccharide (AOS) is a natural non-toxic antioxidant with multiple biological activities. This study analyzed the potential mechanism of AOS in alleviating heat stress and improving semen quality in boars through a combination of multiple omics tools. The results indicated that AOS could significantly increase sperm motility (*P* < 0.001) and sperm concentration (*P* < 0.05). At the same time, AOS improved the antioxidant capacity of blood and semen, and increased blood testosterone (*P* < 0.05) level. AOS could improve the metabolites in sperm, change the composition of gut microbiota, increase the relative abundance of beneficial bacteria such as *Pseudomonas* (*P* < 0.01), *Escherichia-Shigella* (*P* < 0.05), *Bifidobacterium* (*P* < 0.01), reduce the relative abundance of harmful bacteria such as *Prevotella_9* (*P* < 0.05), *Prevotellaceae_UCG-001* (*P* < 0.01), and increase the content of short chain fatty acids. Proteomic results showed that AOS increased proteins related to spermatogenesis, while decreasing heat shock protein 70 (*P* < 0.05) and heat shock protein 90 (*P* < 0.01). These results were verified using immunofluorescence staining technology. There was a good correlation among sperm quality, sperm metabolome, sperm proteome, and gut microbiota. In conclusion, AOS can be used as a feed additive to increase the semen quality of boars to enhance reproductive performance under heat stress.

**Supplementary Information:**

The online version contains supplementary material available at 10.1007/s44154-024-00177-7.

## Introduction

Temperature is a prominent environmental factor that necessitates attention with regard to animal production, since it is widely affecting the growth and reproductive performance of mammals (Kumar et al. 2021; Serviento et al. 2020). When the environmental temperature exceeds the limitation that animals tolerate, the heat stress reaction often occurs (Belhadj Slimen et al. 2016). The main manifestations include increased heat production and sweating. Excessive heat stress can lead to irreversible loss of function (Luo et al. 2021). Swine are animals that are sensitive to temperature. Due to a thick subcutaneous fat layer, underdeveloped sweat glands and poor thermal regulation, the heat generated as a byproduct of a pig’s metabolic processes is difficult to evaporate through the skin (Stombaugh et al. 1973). Research has shown that reproductive traits in pigs are less influenced by genetic factors (Dervishi et al. 2021). On the contrary, they are more influenced by environmental factors (Madsen et al. 2018). Studies have shown that when the average humidity of the pig barn reaches 30% and the average temperature reaches 28 °C, heat stress will occur, which will significantly affect the intestinal health and growth performance of pigs (Serviento et al. 2020; Guo et al. 2018; Wegner et al. 2016), especially for boars and sows with large body weight. The body weight of adult boars is around 300kg (Zhou et al. 2022), and when the ambient temperature exceeds 32 °C and lasts a long time, boars with that weight will undergo heat stress, which can reduce the semen quality (Gruhot et al. 2020). The reason for this is two-fold. On the one hand, in the condition of heat stress, the testicular temperature will also rise, which affects the testicular ability to regulate temperature (Thundathil et al. 2012). On the other hand, sperm stored in the epididymis of a heat-stressed boar produces more radical oxygen species through respiration, resulting in a decline in semen quality (Jannatifar et al. 2019). The ideal breeding environment of a large-scale boar stud has a humidity that cannot exceed 50%, and a temperature that cannot exceed 24 °C in China (Gruhot et al. 2020; Sui et al. 2022). Therefore, adopting nutritional regulation to alleviate the decrease in semen quality caused by heat stress has positive significance for the swine industry.

Alginate is a type of polysaccharide polymer that has been widely used in the pharmaceutical and food industries due to its unique physicochemical properties and beneficial health effects (Mrudulakumari Vasudevan et al. 2021). However, due to the low water solubility and high viscosity of alginate, its development and application are limited. Alginate oligosaccharides (AOS) are degradation products of alginate, characterized by a low molecular weight, high solubility in water and non-toxic nature (Lu et al. 2022), and it has received widespread attention in recent years. AOS has multiple biological activities including immune regulation, anti-inflammatory properties, anti-oxidative effects and even anti-cancer activity. One of the key functions of AOS in immune regulation is to induce cytokine activity. Research had also shown that AOS could increase the concentrations of immunoglobulins and IgA in the serum of weaned piglets, thereby enhancing their immune function (Wan et al. 2018a, b). AOS is a strong antioxidant to scavenge free radicals. Research had found that AOS increased the levels of superoxide dismutase (SOD), and total antioxidant capacity (T-AOC) in the serum and intestine of weaned piglets, while reducing the levels of malondialdehyde (MDA) in the serum and intestine (Wan et al. 2018a, b). AOS could also inhibit the TLR4/NF-κB signaling pathway to enhance the intestinal anti-inflammatory ability of weaned piglets (Wan et al. 2021a, b). In our previous research, we found that AOS could improve the sperm motility of mice that were treated with Busulfan, and repaired the damaged testicular tissue (Zhang et al. 2021). However, there are few reports on AOS in alleviating heat stress and improving boar semen quality. The aim of this investigation was to explore the potential mechanism of AOS in improving semen quality of Duroc boars under heat stress, and to provide a theoretical basis for solving the problem of poor semen quality caused by high temperature in summer.

## Results

### AOS improved boar semen quality under heat stress condition

**Fig. 1:**
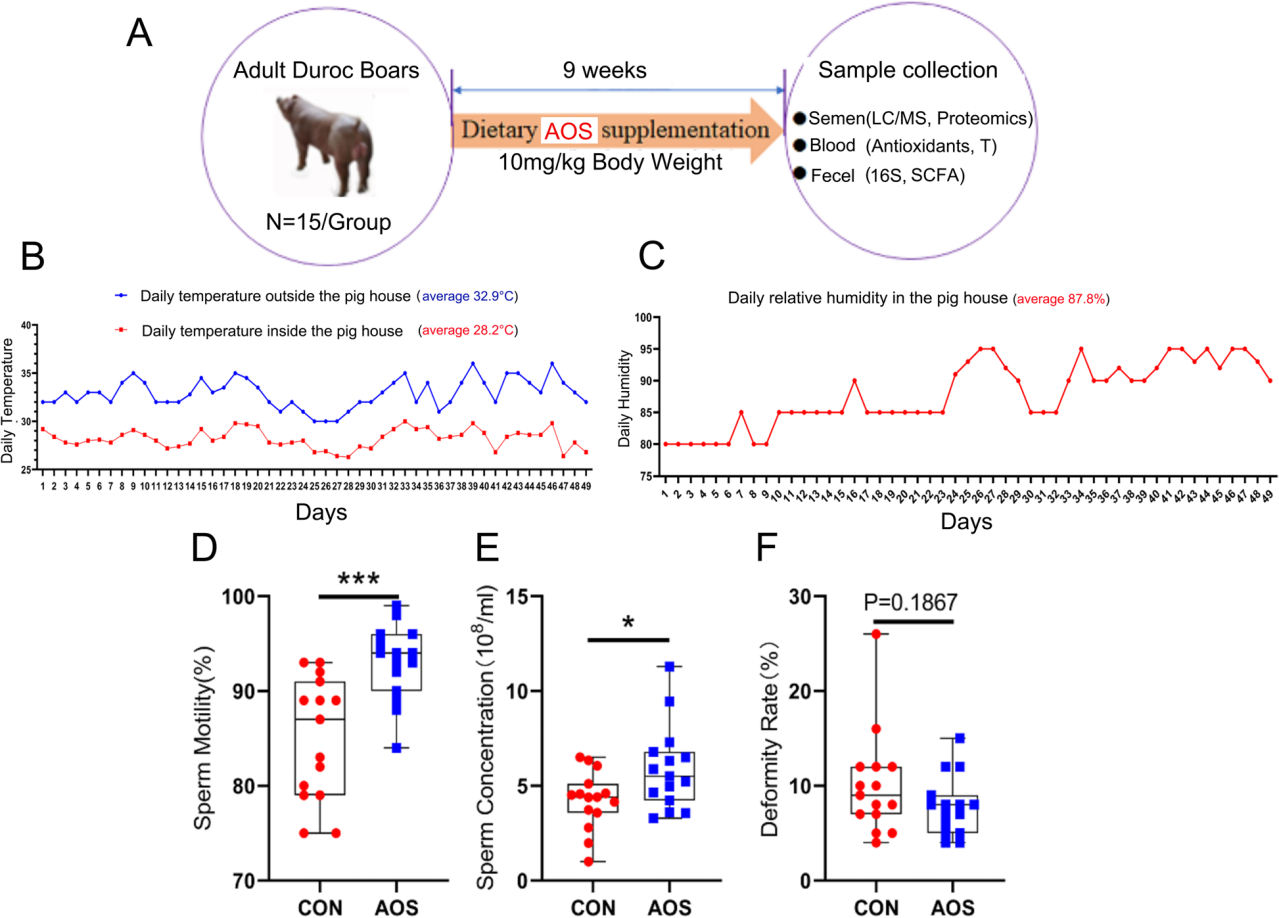
Effects of AOS on the semen quality under heat stress. **A** Study design. **B** Temperature variation. The y-axis represents the temperature. The x-axis represents the number of days during the experiment. The blue line represents outdoor temperature, the red line represents indoor temperature. **C** Humidity variation. The y-axis represents the humidity. The x-axis represents the number of days during the experiment. **D** Sperm motility. The y-axis represents the percentage of total cells. The x-axis represents the treatment (*n* = 15/group). ^***^P < 0.001. **E** Sperm concentration. The y-axis represents concentration. The x-axis represents the treatment (*n* = 15/group). ^*^P < 0.05 (**F**) Abnormal sperm rate. The y-axis represents the percentage of abnormal cells. The x-axis represents the treatment. Data were expressed as the mean ± SEM

As shown in Fig. 1A (Study scheme), thirty adult Duroc boars were fed a control diet (CON group) or a diet supplemented with 10 mg/kg body weight of AOS (AOS group) for nine weeks. Daily temperature and humidity from day 1 to day 49 were recorded. The average temperature outside was 32.9 °C while inside the pig house was 28.2 °C. The average relative humidity was 87.8% (Fig. 1B and C). Feeding AOS was observed to significantly improve boar sperm motility (Fig. 1D; *P* < 0.001) and sperm concentration (Fig. 1E; *P* < 0.05). Moreover, AOS also tended to reduce the abnormal sperm rate (Fig. 1F; *P* = 0.1867). However, there was no difference in abnormal sperm rate between AOS and CON group.

### AOS improved antioxidant indicators and testosterone content in boar plasma

**Fig. 2:**
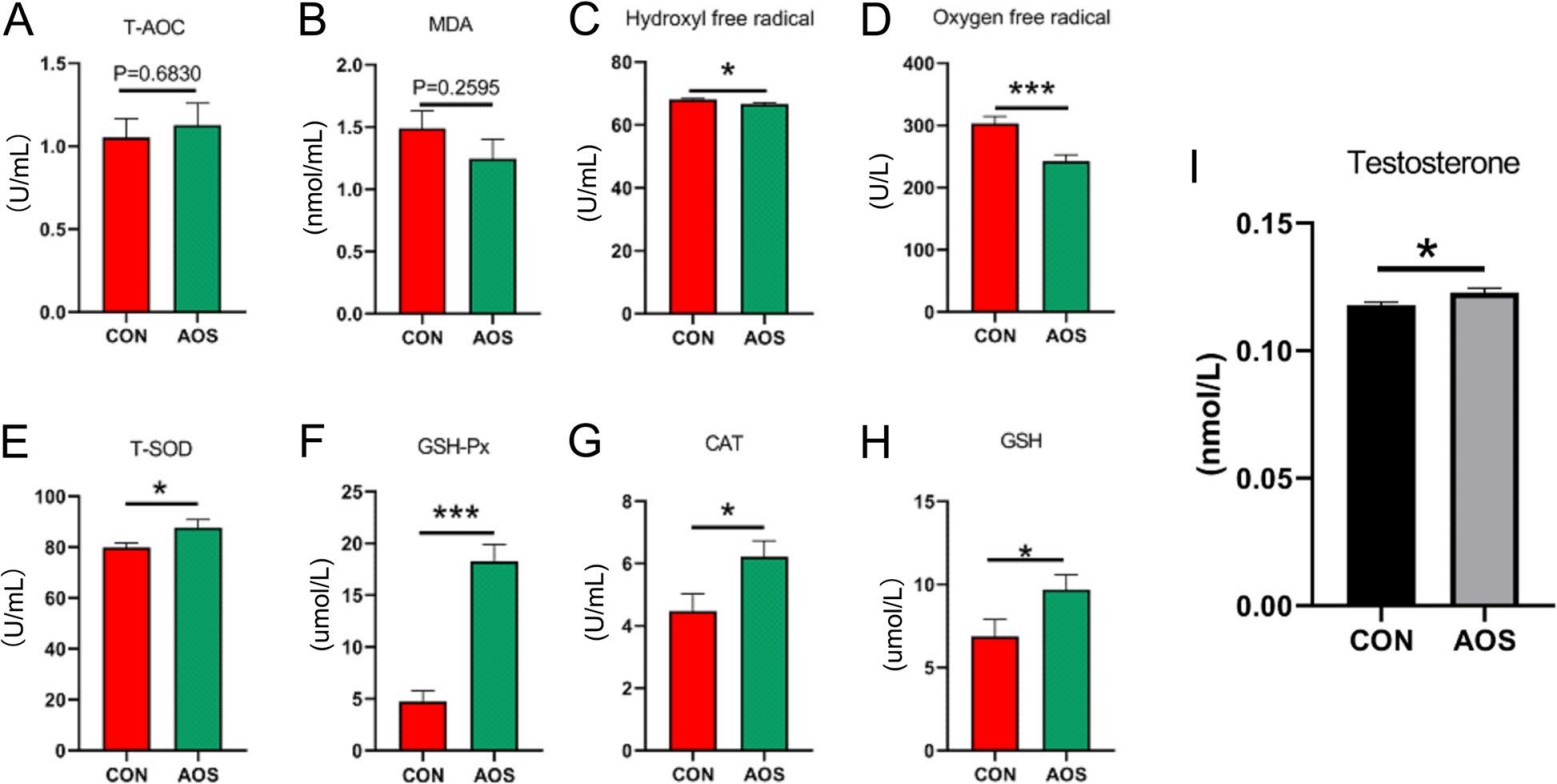
AOS improved antioxidant indicators and testosterone content in boar plasma. **A** T-AOC level. **B** MDA level. **C** Hydroxyl free radical level. **D** Oxygen free radical level. **E** T-SOD level. **F** GSH-Px level. **G** CAT level. **H** GSH Level. **I** testosterone level. Data were expressed as the mean ± SEM. The y-axis represents the amount. The x-axis represents the treatments (*n* = 15/group). ^***^P < 0.001, ^*^P < 0.05

Heat stress is usually accompanied by oxidation stress, so for this reason we tested the antioxidant indicators present in boar blood. From Fig. 2, it can be seen that there were 6 out of 8 different antioxidant indicators that were significantly different between AOS and CON group, namely hydroxyl free radical (Fig. 2C; *P* < 0.05), oxygen free radical (Fig. 2D; *P* < 0.001), total superoxide dismutase (T-SOD) (Fig. 2E; *P* < 0.05), glutathione peroxidase (GSH-Px) (Fig. 2F; *P* < 0.001), catalase (CAT) (Fig. 2G; *P* < 0.05), glutathione (GSH) (Fig. 2H; *P* < 0.05). The T-AOC and MDA levels showed no differences (Fig. 2A and B; *P* > 0.05). AOS can also significantly increase the testosterone content in the plasma (Fig. 2I; *P* < 0.05). The data indicated that AOS could improve the antioxidant capacity of boars to alleviate heat stress and increase important hormone levels related to semen quality in plasma.

### AOS improved the metabolites and antioxidant indicators of sperm

**Fig. 3:**
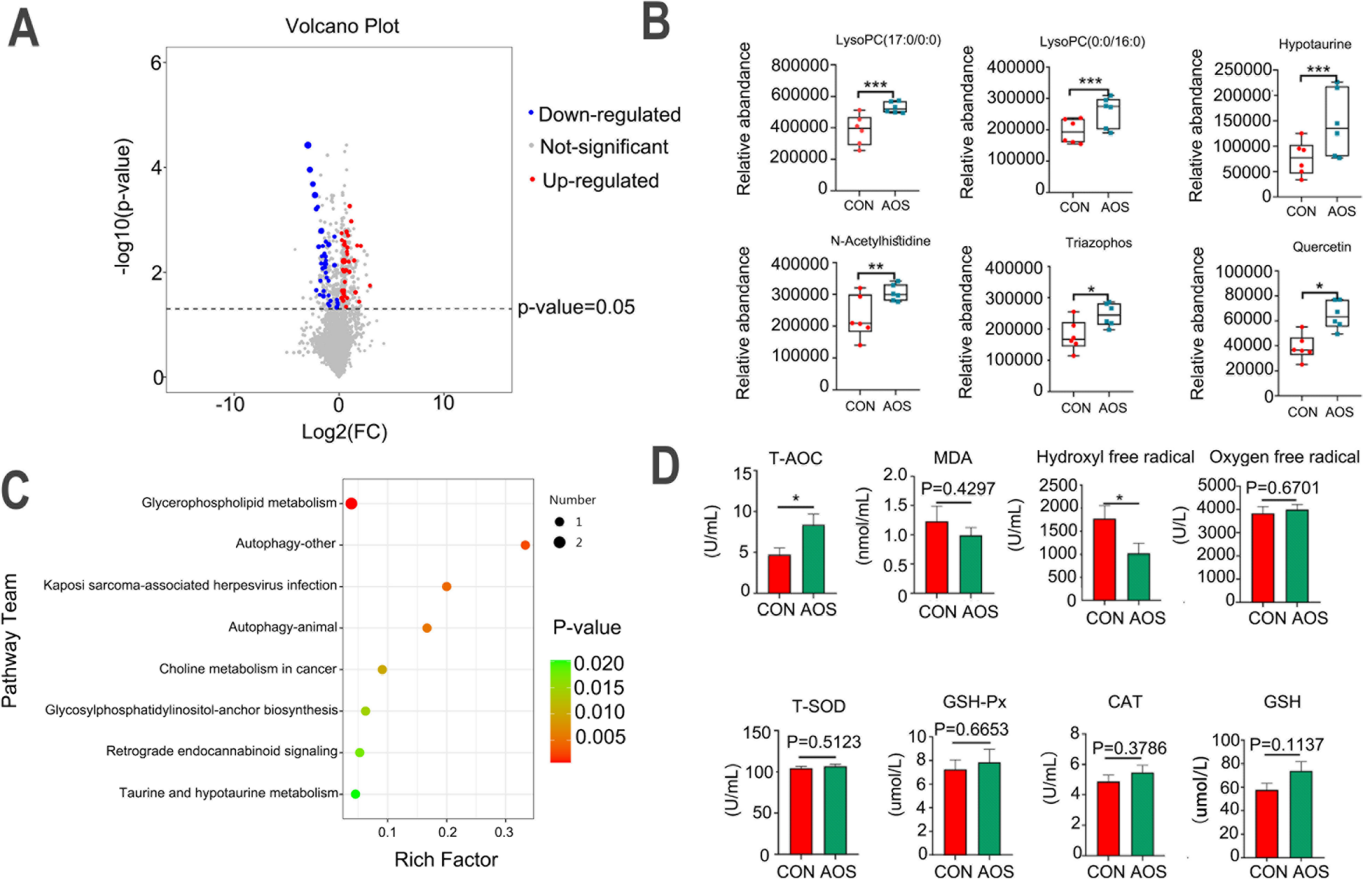
AOS improved the metabolites and antioxidant indicators of sperm. **A** Volcano plot of sperm metabolites. **B** Significant difference metabolites in the sperm including LysoPC (17:0/0:0), LysoPC (0:0/16:0), Hypotaurine, N-Acetylhistidine, Triazophos and Quercetin. **C** The top 8 functional prediction metabolic pathways of sperm metabolites. **D** Sperm antioxidant indicators including T-AOC, MDA, Hydroxyl free radical, Oxygen free radical, T-SOD, GSH-Px, CAT and GSH. Data were expressed as the mean ± SEM. The y-axis represents the relative amount. The x-axis represents the treatments. ^***^P < 0.001. ^**^P < 0.01.^*^P < 0.05

AOS benefited the sperm metabolites which were determined by LC/MS analysis (Table S3). We tested a total of 3361 different metabolites, of which 33 were significantly up-regulated and 35 were significantly down-regulated in the AOS group compared to the CON group (Fig. 3A). Among them, AOS could significantly increase phospholipids in sperm such as LysoPC (17:0/0:0) (Fig. 3B; *P* < 0.001) and LysoPC (0:0/16:0) (Fig. 3B; *P* < 0.001). AOS could also increase amino acids such as Hypotaurine (Fig. 3B; *P* < 0.001) and N-Acetylhistidine (Fig. 3B; *P* < 0.01). Meanwhile, AOS elevated sperm antioxidants such as Triazophos (Fig. 3B; *P* < 0.05) and Quercetin (Fig. 3B;*P* < 0.05). The potential metabolic pathways of the changed metabolites were determined by KEGG pathway analysis (Fig. 3C). AOS was also observed to improve the antioxidant capacity in sperm. The T-AOC level was significantly increased in the AOS group compared to the CON group (Fig. 3D; *P* < 0.05). Meanwhile, AOS significantly reduced Hydroxyl free radical level (Fig. 3D; *P* < 0.05). AOS also benefited other antioxidant indicators, but not significantly (Fig. 3D).

### AOS improved sperm proteome to increase semen quality under heat stress condition

**Fig. 4:**
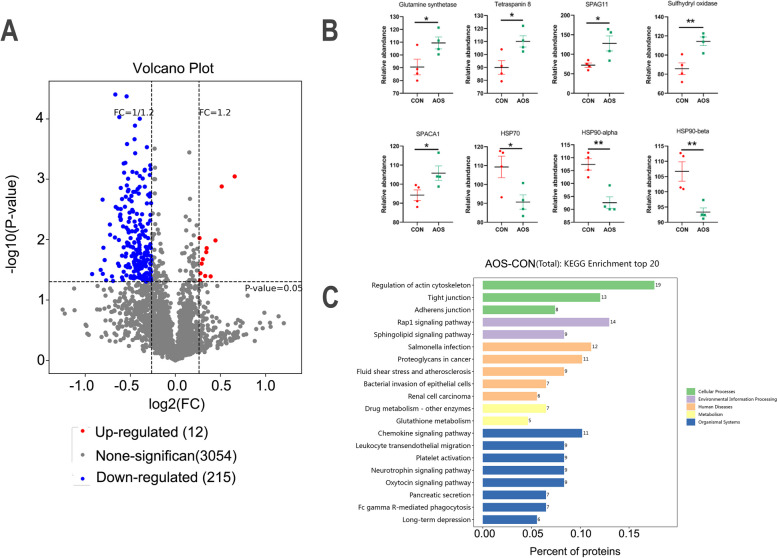
AOS improved sperm proteome to increase semen quality under heat stress condition. **A** Volcano plot of sperm proteomics. **B** Significant difference proteins in the sperm including Glutamine synthetase, Tetraspanin8, Sperm associated antigen11 (SPAG11), Sulfhydryl oxidase, Sperm acrosome membrane-associated protein 1 (SPACA1), Heat shock protein 70 (HSP 70), Heat shock protein 90-α (HSP 90-α) and Heat shock protein 90-β (HSP 90-β). **C** The top 20 functional prediction metabolic KEGG pathways of sperm proteins. Data were expressed as the mean ± SEM. The y-axis represents the relative amount. The x-axis represents the treatments (*n* = 4/group). ^**^P < 0.01.^*^P < 0.05

In order to explore how AOS alleviates heat stress and improves semen quality, we conducted a proteomics analysis. We tested a total of 3281 different proteins, of which 12 were significantly up-regulated and 215 were significantly down-regulated in the AOS group compared to the CON group (Fig. 4A). Among them we found proteins involved in spermatogenesis that were significantly increased such as Glutamine synthetase (Fig. 4B; *P* < 0.05), Tetraspanin 8 (Fig. 4B; *P* < 0.05), Sperm associated antigen11 (SPAG11) (Fig. 4B; *P* < 0.05), Sulfhydryl oxidase (Fig. 4B; *P* < 0.01), and Sperm acrosome membrane-associated protein 1 (SPACA1) (Fig. 4B; *P* < 0.05). Meanwhile, other proteins related to heat stress were significantly decreased such as Heat shock protein 70 (HSP 70) (Fig. 4B; *P* < 0.05), Heat shock protein 90-α (HSP 90-α) (Fig. 4B; *P* < 0.01), and Heat shock protein 90-β (HSP 90-β) (Fig. 4B; *P* < 0.01). The functional prediction of the changed proteins was determined by KEGG pathway analysis (Fig. 4C). Five categories of KEGG terms could be distinguished to differ between the AOS and CON group such as cellular process, environment, human disease, metabolism and organismal system.

### AOS improved the level of proteins related to spermatogenesis under heat stress condition

**Fig. 5:**
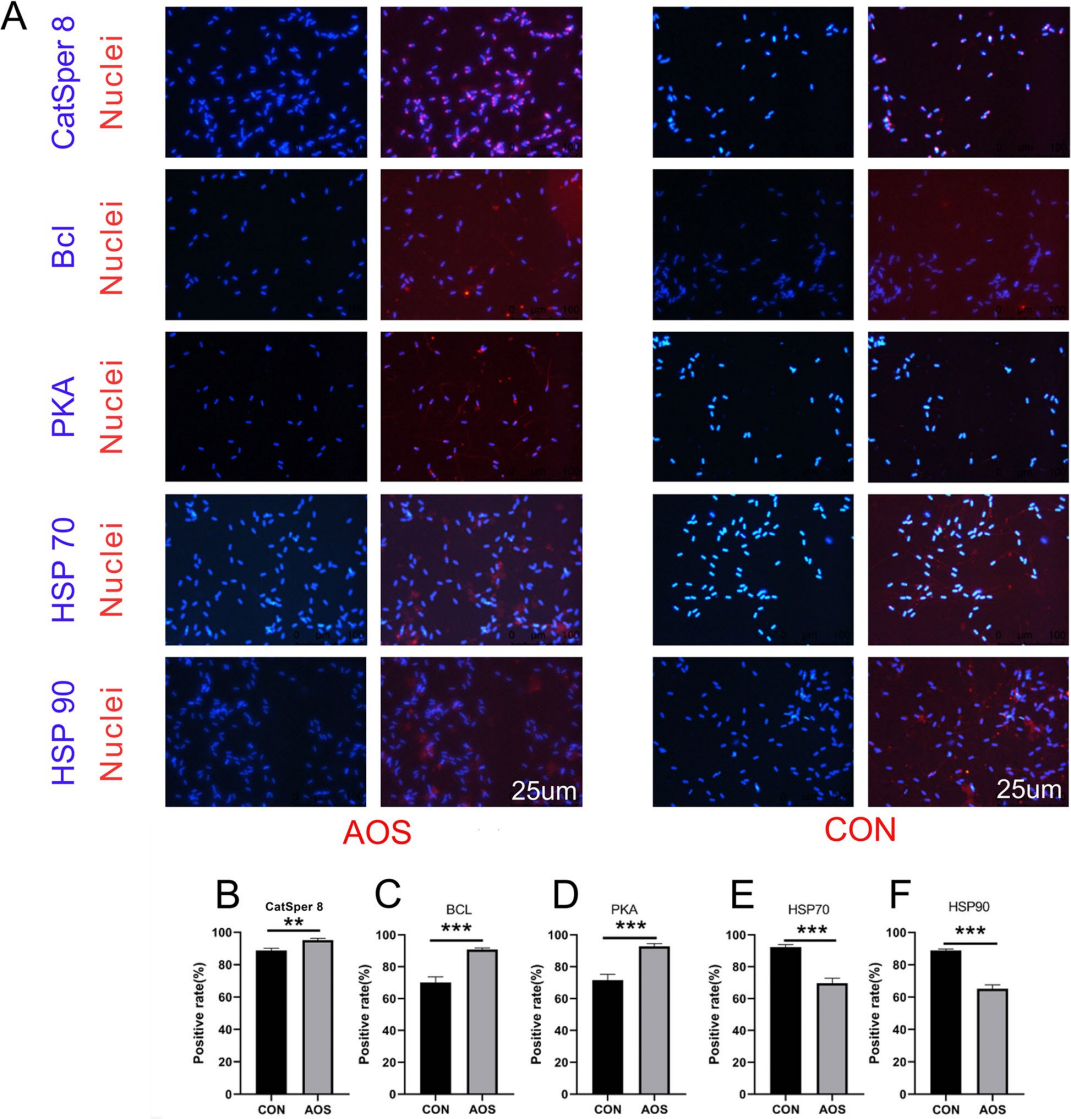
AOS improved the protein level related to spermatogenesis under heat stress condition. **A** Immunofluorescence staining (IHF) of Catsper 8, Bcl, PKA, HSP70 and HSP90. **B** Positive rate of Catsper 8. **C** Positive rate of Bcl. **D** Positive rate of PKA. **E** Positive rate of HSP70. **F** Positive rate of HSP90. Data were expressed as the mean ± SEM. The y-axis represents the amount of Positive rate. The x-axis represents the treatments (*n* = 6/group). ^***^P < 0.001.^**^P < 0.01

To understand how AOS improved boar semen quality, the protein levels of CatSper 8, PKA, Bcl, HSP 70 and HSP 90, all proteins important for sperm quality under heat stress, were quantified (Fig. 5A). Using IHF staining, it could be observed that AOS increased the protein levels of CatSper 8, PKA and Bcl significantly compared to the CON group (Fig. 5B-D; *P* < 0.01). On the other hand, AOS decreased the HSP 70 and HSP 90 level significantly compared to CON group (Fig. 5E-F; *P* < 0.001).

### AOS changed microbial composition and improved SCFAs level in boar feces

**Fig. 6:**
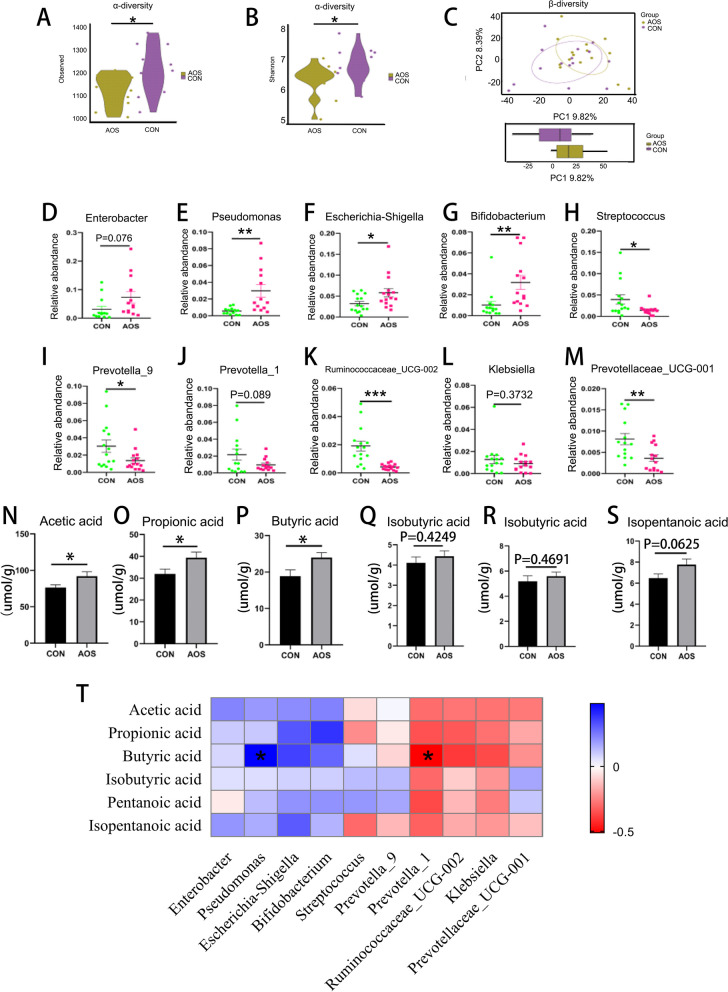
Effects of AOS on the fecal microbial composition and SCFAs. **A** α-diversity with Observed. **B** α-diversity with Shannon. **C** β-diversity with PCoA. The relative amount of individual microbiota in feces at Genus level (**D**-**M**). Data were expressed as the mean ± SEM. The y-axis represents the relative amount. The x-axis represents the treatments (*n* = 15/group). ^***^P < 0.001. ^**^P < 0.01.^*^P < 0.05. **N** Acetic acid level. **O** Propionic acid level. **P** Butyric acid level. **Q** Isobutyric acid level. **R** Pentanoic acid level. **S** Isopentanoic acid level. **T** The correlation between gut microbes and SCFAs. Data were expressed as the mean ± SEM. The y-axis represents the relative amount. The x-axis represents the treatments (*n* = 15/group). ^*^P < 0.05

To investigate the effect of AOS on boar intestinal microbes, we conducted 16 s sequencing and short-chain fatty acids determination in feces. The α-diversity measured using the observed taxa metric (Fig. 6A; *P* < 0.05) or using the Shannon index (Fig. 6B; *P* < 0.05) was significantly different. The microbiota composition was also observed to be different between AOS and CON group by PCA analysis (Fig. 6C). AOS increased the abundance of beneficial microbiota at genus level such as *Enterobacter* (Fig. 6D; *P* = 0.076), *Pseudomonas* (Fig. 6E; *P* < 0.01), *Escherichia-Shigella* (Fig. 6F; *P* < 0.05), and *Bifidobacterium* (Fig. 6G; *P* < 0.01). At the same time, AOS decreased the abundance of harmful microbiota such as *Streptococcus* (Fig. 6H; *P* < 0.05), *Prevotella_9* (Fig. 6I; *P* < 0.05), *Prevotella_1* (Fig. 6J; *P* = 0.089), *Ruminococcaceae_UCG-002* (Fig. 6K; *P* < 0.001), *Klebsiella* (Fig. 6L; *P* = 0.3732), and *Prevotellaceae_UCG-001* (Fig. 6M; *P* < 0.01). To analyse the metabolites of gut microbes, we measured the SCFAs in the feces. Acetic acid (Fig. 6N; *P* < 0.05), propionic acid (Fig. 6O; *P* < 0.05), butyric acid (Fig. 6P; *P* < 0.05) were significantly increased in the feces after feeding AOS, others, such as isobutyric acid (Fig. 6Q; *P* = 0.4249), pentanoic acid (Fig. 6R; *P* = 0.4691), isopentanoic acid (Fig. 6S; *P* = 0.0625), were also increased but not significantly. The correlation between gut microbiota and SCFA is shown in (Fig. 6T). Butyric acid had a strong positive correlated with the beneficial bacterium *Pseudomonas* (*P* < 0.05) and a strong negative correlated with the harmful bacterium *Prevotella_1* (*P* < 0.05).

### Spearman correlation among fecal microbes, sperm protein, sperm metabolites and sperm parameters

**Fig. 7:**
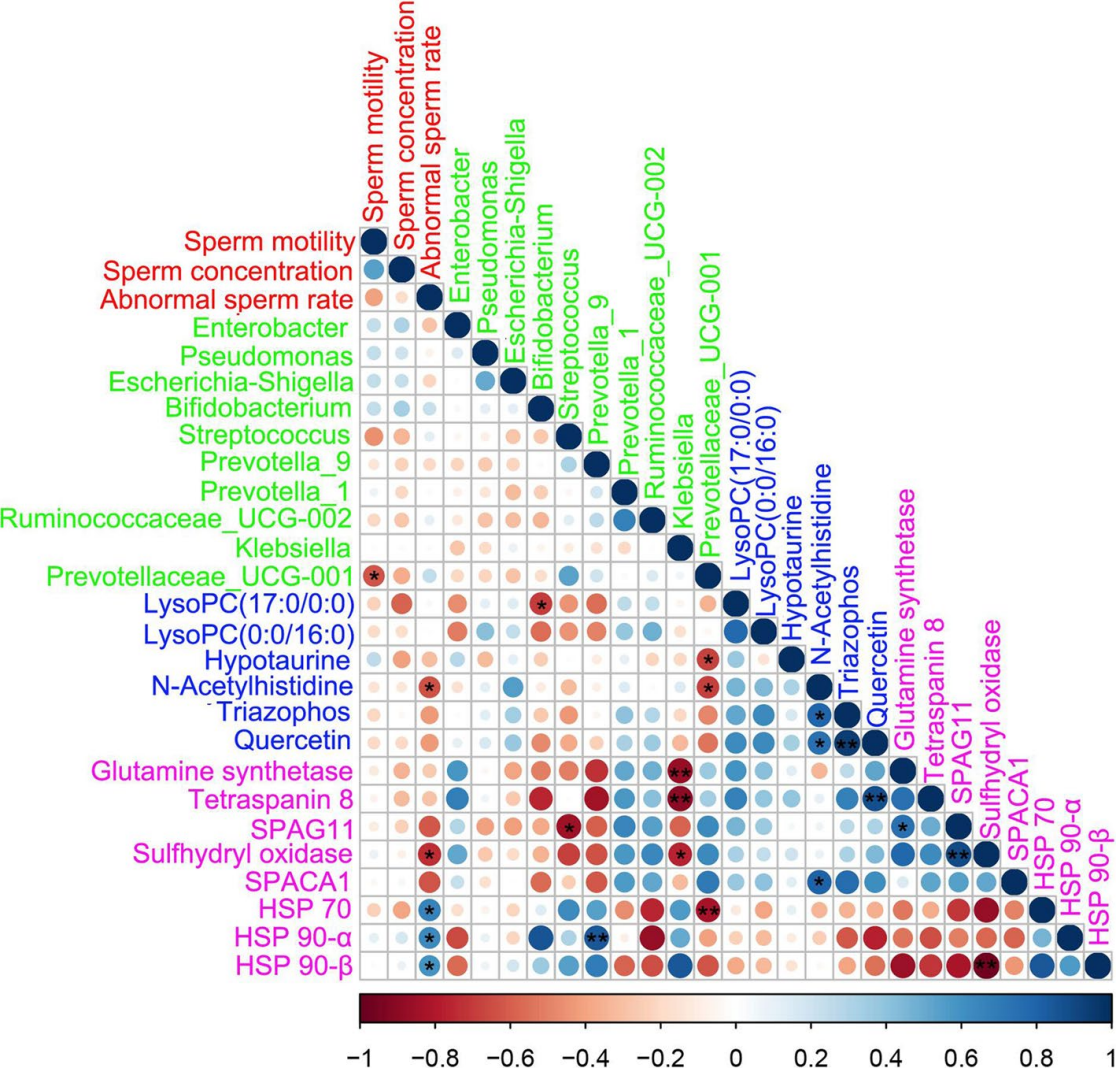
Correlations. Correlations among fecal microbes, sperm metabolites, sperm protein and semen quality parameters. Color red represents sperm parameters, color green represents gut microbes, color blue represents sperm metabolites, color pink represents sperm protein. Blue cycles represent positive correlation, red cycles represent negative correlation. The size of the circle represents the strength of the correlation. (larger circle = stronger correlation). ^**^P < 0.01.^*^P < 0.05

The spearman correlation analysis (Fig. 7) indicated that the fecal microbiota, sperm metabolites, sperm protein and semen parameters were well correlated. Firstly, there was a significant negative correlation between sperm motility and the harmful bacterium *Prevotellaceae_UCG-001*. Next, the abnormal sperm rate was significantly negatively correlated with the N-acetylhistidine and sulfhydryl oxidase. At the same time, the abnormal sperm rate was positively correlated with the level of heat shock proteins. In addition, there was also a good correlation between fecal microorganisms, sperm metabolites and the sperm proteome.

## Discussion

Alginate Oligosaccharide (AOS) is an oligosaccharide containing 2 ~ 25 sugar units, which is obtained from the cleavage of algin at the glycosidic bond or the biosynthesis of its monosaccharide components (Lu et al. 2022). Due to its low molecular weight, high water solubility, safety and non-toxic characteristics (Wang et al. 2021), AOS has received high attention from researchers in recent years. AOS has a variety of unique biological activities, such as immune regulation (Bland et al. 2004), antioxidative properties (Falkeborg et al. 2014), and anti-cancer activities (Liu et al. 2022). AOS holds significant promise and is currently a focal point of research in several fields including drug development, nutritional health and sustainable agriculture (Pritchard et al. 2017). Reactive oxygen species (ROS), such as oxygen or hydroxyl free radicals, are substances produced by the metabolism under normal physiological and pathological conditions (Su et al. 2019). Excessive accumulation of ROS may however cause harmful oxidative stress, and induce structural (Valko et al. 2007) damages in cells, including harm to proteins (Hawkins and Davies 2019) and DNA (Marnett 2000). Excessive oxidative damage can lead to loss of cell function and ultimately result in cell apoptosis (Nordberg and Arnér 2001). In this study, we found that the content of reactive oxygen species (oxygen free radicals and hydroxyl radicals) in boar blood decreased significantly by feeding AOS, while, at the same time, the content of hydroxyl radicals in boar sperm also decreased significantly. This indicated that AOS could alleviate the degree of oxidative stress caused by heat stress to potentially improve semen quality by reducing the reactive oxygen species in blood and sperm.

Heat stress refers to the sum of non-specific physiological reactions that the body undergoes in a hot environment at high environmental temperatures (Cramer et al. 2022). Heat stress can disrupt antioxidant systems such as SOD (Liu et al. 2021), CAT (Ouyang et al. 2022), and GSH-px (Chang et al. 2022). Research had shown that in boars exposed to 40 °C for 5 h per day for 8 days, the MDA level was significantly increased in the epididymis, indicated that heat stress caused significant oxidative stress damage to testicular tissue (Li et al. 2017). In this study, it was found that the antioxidant indicators SOD, GSH-px, and CAT content in boar serum were significantly increased, while the T-AOC content in boar sperm was also significantly increased, indicating that AOS exerted a strong antioxidant ability. In other studies, it has been found that AOS could alleviate the damage caused by oxidative stress (Saghir et al. 2023), while improving sperm motility of breeding boars (Han et al. 2022). This is consistent with our experimental results. Therefore, we inferred that AOS could alleviate the adverse effects of heat stress on semen quality by exerting strong antioxidant properties. Testosterone in blood can improve sexual desire (van Anders 2012), which is an important male hormone to promote spermatogenesis (Smith and Walker 2014). High temperature and high humidity environments can easily lead to a decrease in sexual desire and sperm quality in boars (Chen et al. 2018). However, in this experiment, the testosterone content in the blood was significantly increased by adding AOS. Therefore, AOS could improve the testosterone level under heat stress conditions, thereby positively influencing semen quality.

Several proteins are significant raw material for spermatogenesis (Chalmel and Rolland 2015). Studies have shown that glutamine synthetase can synthesize glutamine, which is an amino acid proven to affect mammalian sperm motility (Francou et al. 2012). Tetraspanin 8 is a protein critical to male fertility and found in sertoli cells in the testis (Pradhan et al. 2020). SPAG affects sperm vitality of mammals. Lack of SPAG will lead to abnormal spermatogenesis, which greatly increases the abnormal sperm rate (Liu et al. 2019; Sangeeta and Yenugu 2022). SPACA1 is a protein that affects the integrity of the sperm acrosome and plays an important role in the fertilization process (Minami et al. 2020). In this study, we conducted a proteome analysis on boar sperm and found that supplementing diet with AOS significantly improved the protein levels of the aforementioned proteins under heat stress conditions. At the same time, the levels of heat shock protein 70 and heat shock protein 90 significantly decreased. Research has shown that when animals are exposed to high temperatures for a long time, they synthesize heat shock proteins in order to protect themselves (Goto et al. 2022; Thirumalaikumar et al. 2021). We validated the proteomic results by using IHF and found consistent results. In addition, the content of other proteins related to spermatogenesis increased significantly, such as CatSper 8 (Rahban et al. 2021), PKA (Baro Graf et al. 2020) and Bcl (Cayli et al. 2004). The data indicated that AOS could increase proteins related to spermatogenesis and reduce the content of heat shock proteins, thus alleviate the adverse effects of heat stress on boar semen quality.

In this study, in order to investigate the changes of sperm metabolites under heat stress, we determined the metabolome of boar sperm. We could find that after supplementing with AOS, there were notable improvements in specific sperm metabolites, mainly phospholipids, amino acids and antioxidants, indicating a significant positive change. Previous studies had reported that LysoPC (17:0/0:0) and LysoPC (0:0/16:0) exerted a potential protective role in regulating metabolic disorders (Zhang et al. 2020). Hypotaurine is an amino acid that can be used to improve the post-thawed Merino ram sperm parameters (Bucak et al. 2013). Triazophos is a strong antioxidant that is used in previous research and led to substantial up-regulation of a male spermatogenesis-associated protein 5-like gene (NlSPATA5) to increase semen quality (Ge et al. 2016). Quercetin is widely reported in many fields, and it can increase testosterone and sperm concentration in mice (Garcia et al. 2023). Quercetin can also ameliorate sperm oxidative stress and inflammation, while preserving sperm morphology and function in rats (Yelumalai et al. 2019). Therefore, AOS can increase the antioxidant content and change the amino acid level in semen to benefit spermatogenesis.

The intestine is the largest digestive organ (Gasbarrini et al. 2008) of the animal body. Gut microbiota is the intermediary between host and diet, thus it plays a vital role in regulating animal health (Bibbò et al. 2016). Research showed that plant extracts can improve the fecundity of male animals by changing their gut microbiota (Zhou et al. 2022). In our previous studies we found that AOS can improve the semen quality of boars and mice by improving gut microbiota and sperm metabolites (Yan et al. 2022; Han et al. 2022). In this study, we conducted 16s sequencing analysis on boar feces, and examined enriched microbiota species at the genus level. It was found that the relative abundance of four beneficial bacteria related to spermatogenesis increased, while the relative abundance of six harmful bacteria decreased. Among them, the beneficial bacterium *Enterobacter* can improve the storage time of boar semen at room temperature (Prieto-Martínez et al. 2014). *Pseudomonas* was beneficial for sperm capacitation and protein phosphorylation of boar spermatozoa (Sepúlveda et al. 2016, 2014). *Escherichia-Shigella* was positively correlated with the synthesis of sex hormones (Ramírez-Acosta et al. 2022). *Bifidobacterium* can be used for patients with asthenozoospermia for 6 weeks, after which semen quality is significantly improved (Valcarce et al. 2017). Meanwhile, *Prevotella* is considered a harmful bacterium in terms of affecting male fertility. Through the study of semen microbial composition and male fertility, it has been noted that *Prevotella* has a negative impact on sperm motility and concentration (Farahani et al. 2021; Lundy et al. 2021; Gachet et al. 2022). *Streptococcus* and *Klebsiella* were the most susceptible bacteria during fertilization, reducing sperm motility and ultimately leading to sperm apoptosis (Zuleta-González et al. 2019). In order to further explore the relationship between gut microbiota and semen quality, we measured short-chain fatty acids in boar feces. Short-chain fatty acids mainly participate in the energy supply of intestinal epithelial cells (Hu et al. 2018) and affecte the permeability of intestinal mucosa (Usuda et al 2021). At present, more and more studies indicated the existence of an intestinal-testicular axis (Hao et al. 2022). Gut microbiota can use carbohydrates to produce short chain fatty acids (Chen et al. 2019; He et al. 2020). A correlation analysis study pointed out that SCFAs could improve semen quality (Lin et al. 2022). A study by Olaniyi et al. has also shown that SCFAs significantly decreased testicular proprotein convertase subtilisin/kexin type 9 (PCSK9) with a significant reduction in cholesterol and lipid peroxidation as a result. This reduction resulted in enhanced testicular tissue, characterized histologically by the restoration of tissue architecture, seminiferous tubules and spermatogonia, along with improved testicular function, including spermatogenesis and steroidogenesis (Olaniyi et al. 2021). In this study, the addition of AOS significantly increased the content of acetic acid, propionic acid, and butyric acid, and there was a significant positive correlation between beneficial bacteria *Pseudomonas* and butyric acid content, while harmful bacteria *Prevotella* had a significant negative correlation with butyric acid production. The above data indicated that the potential mechanism of AOS to improve boar semen quality may be to increase the relative abundance of beneficial bacteria in the gut, that on their turn could produce short-chain fatty acids beneficial for spermatogenesis.

## Materials and methods

### Boars and experimental design

The study was approved by the Animal Care and Use Committee of the Institute of Animal Sciences of Chinese Academy of Agricultural Sciences (access number of IAS2022-13). Thirty Duroc boars with similar age (33 months old), health status and weight (around 300 kg) were selected randomly at the Ya Ji Mountion boar stud which belongs to the Yangxiang Joint Stock Company. The boars were divided into 2 groups randomly, a control group (CON) and AOS group (AOS). The control group (CON) was fed a basal diet (Feed formula is shown in Table S1), and boars in the AOS group (AOS) were fed a basal diet with 10 mg/kg body weight AOS (Han et al. 2022). The boars were housed in individual pens and the whole feeding period lasted 9 weeks. During the experiment, the everyday temperature and humidity were recorded at the same time each day (8:00 am and 14:00 pm).

Semen samples were obtained using the hand grip method. Sperm parameters including sperm concentration, sperm motility and abnormal sperm rate were assessed by Computer-assisted sperm analysis (CASA) software according to previous reports (Guo et al. 2020). A sample of semen was stored at -80 °C for further analyses. During ejaculating, when the boars are immobilized, blood samples were taken from the hind legs. Blood plasma and blood cells were separated by centrifugation at 3000 × g for 10 min, after which plasma was transferred to -80 °C for further testosterone analyses. Fecal samples were taken from the rectum, placed in liquid nitrogen, and finally stored in a -80 °C freezer for 16S analysis and short chain fatty acids determination.

### Sperm parameters analyzed by computer-assisted sperm analysis system (CASA)

Sperm parameters, including sperm concentration, sperm motility, and abnormal sperm rate, were recorded by a computer assisted sperm analysis (CASA) system (Shanghai Kasu Biotechnology Co., Ltd., Shanghai, China). The evaluated criteria of sperm motility was as follows: grade A fast forward movement > 22 μm s^−1^; grade B forward movement < 22 μm s^−1^; grade C curve movement < 5 μm s^−1^; grade D no movement. The sperm concentration should be more than 10^8^/ml, the abnormal sperm rate should be less than 30% (Cao et al. 2011).

### Detection of plasma and sperm antioxidant indicators and blood testosterone content

Antioxidant parameters (T-AOC, SOD, GSH-Px, CAT, MDA, GSH, Hydroxyl free radical, Oxygen free radical) in serum and sperm as well as blood testosterone were tested using ELISA kits (Nanjing Jiancheng Bioengineering Institute, Nanjing, China and Beijing Boxbio Science&Technology Co.,Ltd, respectively) following the manufacturer’s instructions.

### Sperm metabolome assay by LC–MS/MS

Boar sperm (*n* = 6 per group) was taken out from -80 °C freezer. The protein fraction was removed and then the samples were analyzed by LC/MS. An ACQUITY UPLC BEH C18 column (1.7 μm, 2.1 × 100 mm) was employed in both positive and negative modes. Solvent A and solvent B, aqueous solutions containing 0.1% formic acid and 0.1% acetonitrile respectively, were used. The following procedure was performed: 5–20% B over 0–2 min; 20–60% B over 2–4 min; 60–100% B over 4–11 min, keep the composition at 100% B for 2 min, 13–13.5 min from 100 to 5% B, and hold it at 5% B for 13.5–14.5 min. The flow rate was set at 0.4 mL/minute and the column temperature was 45 °C. The sperm was stored at 4 °C and each time 5 μL was used to inject. ESI was used in the mass spectrometry program.

### Quantitative analysis of boar sperm proteome using TMT labeling

Frozen samples (*n* = 4 per group) were transferred into low protein binding tubes and lysed with 300 μL lysis buffer supplemented with 1mM PMSF. Then samples were further lysed with sonication. The parameters were set as 1s /1s intervals and 80 W for 2 min. After sonication, the samples were centrifuged at 12000 rpm for 10 min at 4 °C to remove insoluble particles, and this was repeated once to further exclude precipitation. Protein concentration was determined by BCA assay. The protein samples were aliquoted to store at -80 °C.

According to the measured protein concentration, the same quality protein was taken from each sample, and different groups of samples were diluted to the same concentration and volume. Twenty-five mM DTT of corresponding volume was added into the above protein solution to make the DTT final concentration about 5mM, after which this was incubated at 55 °C for 30 min. Then, the corresponding volume of iodoacetamide was added to reach a final concentration of 10mM, after which the solution was placed in the dark for 15 min at room temperature. To precipitate the protein, 6 times of the volume of pre-cooled acetone was added and the sample was placed at -20 °C for more than four hours or overnight. Subsequently, the sample was centrifuged at 8000g for 10 min at 4 °C to collect the precipitate. With respect to the amount of protein, the corresponding volume of enzymolysis diluent (protein: enzyme = 50:1 (m/m), 100 ug of protein and 2 ug of enzyme) was added to redissolve the protein precipitate. The solutions were incubated for digestion at 37 °C for 12 h. Finally, samples were lyophilized or evaporated after enzymolysis.

For TMT pro labelling, the lyophilized samples were resuspended in 100 μL 100 mM TEAB (pH8.5) and 40 μL of each sample were transferred into a new tube for labeling. Anhydrous acetonitrile was added to the TMT reagent vial at room temperature. The centrifuged reagents were dissolved for 5 min and mixed for centrifugation and this step was once repeated. Then 10 μL of the TMT pro label reagent was added to each sample for mixing. The composition was incubated at 17 °C for 1 h. Five μL of 5% hydroxylamine was added to each sample and incubated for 15 min to quench the reaction. The labeling peptide solutions were lyophilized and kept at -80 °C conditions.

The solutions were supplemented with 1% formic acid (FA) to adjust the pH value to 1–3. We utilized a C18-Reverse-Phase SPE Column to desalt the digested peptides. First of all, 1 mL methanol was used to wash the column twice, followed by 0.1% TFA/H2O for 2–3 times. All the samples were loaded on the column. Next, 0.1%TFA/H2O was used to wash the column 3 times. Finally, the peptides were eluted with 90% ACN/H2O (containing 0.1%TFA) 3 times and lyophilized.

Separations were performed on an Agilent Zorbax Extend-C18 column on a 1100 HPLC system. Mobile phases A and B were used for gradient which was set as follows: 98% A over 0–8 min; 98% ~ 95% A over 8–8.01 min;95% ~ 75% A over 8.01–48 min;75 ~ 60% A over 48–60 min; 60 ~ 10% A over 60–60.01 min; 10% A over 60.01–70 min; 10 ~ 98% A over 70–70.01 min and98% A over 70.01–75 min. Tryptic peptides were separated at a fluent flow rate of 300 μL/min and monitored at 210 nm. Samples were collected for 8–60 min, and eluent was collected in centrifugal tubes every minute for 15 min. Samples were recycled in this order until the end of gradient. The separated peptides were lyophilized for mass spectrometry.

### Analysis of protein levels in boar sperm using immunofluorescence staining (IHF)

The IHF methods for boar sperm has been reported in our previous article (Zhou et al. 2022). The IHF analyses was done on the sperm samples of all 30 Duroc boar. Primarily, the boar sperm was fixed in 4% paraformaldehyde for 1 h, then air-dried and spread on slides covered with poly-L-lysine. The slides were washed 3 times with PBS (each time for 5 min), and kept in a container with 2% Triton X-100 in PBS to incubate for 1 h at room temperature. Next, they were washed again 3 times (each time for 5 min) with PBS, after which the sperm was blocked with PBS containing 1% BSA and 1% goat serum for 30 min at 17 °C. The slides were then incubated with a diluted primary antibody (1:100; Table S2) overnight at 4 °C. The next morning, they were washed three times with PBS which contained 1% BSA, each time for 5 min after which the secondary antibody (1:100) was added. Slides were subsequently incubated at 37 °C in the dark for 1 h. After three times washing with PBS solution, Hoechst 33,342 was added in order to stain the nucleus of the sperm for 5 min at room temperature. Finally, the slides was again washed three times with PBS for 5 min each time, after which an accelerator was added and pictures were taken under a fluorescence microscope (LEICA TCS SP5 II, Germany). The protein positive rate was defined as red sperm/total sperm × 100% in the randomly selected view. Each slide was screened 5 times and the resulting average was used to determine the positive rate.

### Boar feces microbiota sequencing and short chain fatty acids determination

The protocol for the analysis of fecal microbiota was reported in our previous study (Zhou et al. 2022) (*n* = 15 per group). An E.Z.N.A. ® Stool DNA Kit (Omega Bio-tek Inc., USA) was used to separate total genomic DNA from feces of boars, followed the manufacturer’s instructions. NanoDrop 2000 (Thermo Scientific, USA) was used to detect DNA quantity and 1% agarose gel was made to test DNA quality. The primers 338F (5'-ACTCCTACGGGAGGCAGCAG-3') and 806R (5'-GGACTACHVGGGTWTCTAAT-3') were used to amplify the V3–V4 region of the microbial 16S rRNA genes. The PCR was done as described in our previous study (Wan et al. 2021a, b). The PCR amplification products were extracted and purified from a 2% agarose gel using the AxyPrep DNA Gel Extraction Kit (AXYGEN, New York, NY, United States). Finally, the sequences were assigned to operational taxonomic units (OTU). The rule was that similar sequences were assigned to an OTU when the similarity level was more than 97%.

GC–MS was used to test the concentration of SCFAs in the feces. One g of feces was weighed into a 1.5 ml centrifuge tube, 1 ml of double-distilled water was added and the sample was subsequently centrifuged at 10000 rpm for 10 min in a low-temperature centrifuge. The supernatant and metaphosphoric acid were mixed in a ratio of 9:1, and centrifuged at 1000 rpm for 10 min at 4 °C for more than two hours. A 0.45 μm filter membrane was used for filtration and the Agilent 6890 gas chromatography was used to analyse the SCFAs (Agilent Technologies, Inc., Palo Alto, CA, United States).

### Statistical analysis

All data were expressed as the mean ± SEM. *P* < 0.05 was considered as significantly different. Student’s t-test was performed for the statistical analyses. Spearman’s correlation analysis was completed by RStudio (version 4.0.3) platform. The plots were made using GraphPad Prism 8.0.2.

## Conclusion

Current research showed that AOS improved boar semen quality during heat stress by improving gut microbiota, sperm metabolome and sperm proteome. Therefore, AOS can be used as a feed additive to solve the problem of decreased semen quality in boars caused by high temperature and humidity in summer.

## Supplementary Information

Supplementary Material 1.

Supplementary Material 2.

Supplementary Material 3.

Supplementary Material 4.

Supplementary Material 5.

Supplementary Material 6.

## Data Availability

The datasets analyzed during the current study are available from the corresponding author upon reasonable request. The raw data of 16s RNA sequencing had been uploaded to the NCBI SRA database with accession number PRJNA1083913.

## Abbreviations

AOS: Alginate oligosaccharide
CASA: Computer-assisted sperm analysis
T-AOC: Total antioxidant capacity
SOD: Superoxide dismutase
GSH-Px: Glutathione peroxidase
CAT: Catalase
MDA: Malondialdehyde
GSH: Glutathione
IHF: Immunofluorescence staining
SPAG11: Sperm associated antigen 11
SPACA1: Sperm acrosome membrane-associated protein 1
HSP 70: Heat shock protein 70
HSP 90-α: Heat shock protein 90-α
HSP 90-β: Heat shock protein 90-β
PKA: Protein kinase A
