# Supplementary material for: Alginate oligosaccharide supplementation improves boar semen quality under heat stress
Source: Stress Biol. 2024 Sep 3;4(1):37. doi: 10.1007/s44154-024-00177-7 (PMC11383898; doi:10.1007/s44154-024-00177-7)
Supplement: Supplementary file 2 — Supplementary Material 2. [file 44154_2024_177_MOESM2_ESM.docx]

**Table S2.** Primary antibody information

| **Name** | **Cat. #** | **Source (Animal)** | **Company** |
| --- | --- | --- | --- |
| Bcl | bs-0520R | Rabbit (polyclonal) | Beijing Biosynthesis Biotechnology CO. |
| PKA | bs-1645R | Rabbit (polyclonal) | Beijing Biosynthesis Biotechnology CO. |
| Anti-HSP 70 | bs-0126R | boar | Beijing Biosynthesis Biotechnology CO. |
| Anti-HSP 90 | Bs-10100R | boar | Beijing Biosynthesis Biotechnology CO. |
| CATSPER | bs-23326R | Rabbit | Beijing Biosynthesis Biotechnology CO. |
